# TTF-1 is a highly sensitive but not fully specific marker for pulmonary and thyroidal cancer: a tissue microarray study evaluating more than 17,000 tumors from 152 different tumor entities

**DOI:** 10.1007/s00428-024-03926-1

**Published:** 2024-10-08

**Authors:** Katharina Möller, Tayyaba Gulzar, Maximilian Lennartz, Florian Viehweger, Martina Kluth, Claudia Hube-Magg, Christian Bernreuther, Ahmed Abdulwahab Bawahab, Ronald Simon, Till S. Clauditz, Guido Sauter, Ria Schlichter, Andrea Hinsch, Simon Kind, Frank Jacobsen, Eike Burandt, Nikolaj Frost, Martin Reck, Andreas H. Marx, Till Krech, Patrick Lebok, Christoph Fraune, Stefan Steurer

**Affiliations:** 1 Institute of Pathology, University Medical Center Hamburg-Eppendorf, Martinistr. 52, 20246 Hamburg, Germany; 2 Department of Basic Medical Sciences, College of Medicine, University of Jeddah, Jeddah, Saudi Arabia; 3 Department of Infectious Diseases and Respiratory Medicine, Charité-Universitätsmedizin Berlin, Corporate Member of Freie Universität Berlin, Humboldt-Universität Zu Berlin, and Berlin Institute of Health, Berlin, Germany; 4 Lung Clinic Grosshansdorf, Airway Research Center North, German Center of Lung Research, Grosshansdorf, Germany; 5 Department of Pathology, Academic Hospital Fuerth, Fuerth, Germany; 6 Institute of Pathology, Clinical Center Osnabrueck, Osnabrueck, Germany

**Keywords:** TTF-1, Immunohistochemistry, Tissue microarray, Diagnosis, Pulmonary adenocarcinomas

## Abstract

Thyroid transcription factor 1 (TTF-1) immunohistochemistry (IHC) is routinely used for the distinction of primary pulmonary adenocarcinomas. However, TTF-1 can also occur in other malignancies. A tissue microarray containing 17,772 samples from 152 different tumor types was analyzed. Napsin-A, CK20, SATB2, FABP1, and Villin-1 IHC data were available from previous studies. TTF-1 staining was seen in 82 of 152 tumor categories including thyroidal cancers (19–100%), adenocarcinomas (94%), neuroendocrine tumors (67%) of the lung, small cell neuroendocrine carcinomas (71–80%), mesenchymal tumors (up to 42%), and thymomas (39%). Comparative analysis of TTF-1 and Napsin-A revealed a sensitivity/specificity of 94%/86% (TTF-1), 87%/98% (Napsin-A), and 85%/99.1% (TTF-1 and Napsin-A) for the distinction of pulmonary adenocarcinomas. Combined analysis of TTF-1 and enteric markers revealed a positivity for TTF-1 and at least one enteric marker in 22% of pulmonary adenocarcinomas but also a TTF-1 positivity in 6% of colorectal, 2% of pancreatic, and 3% of gastric adenocarcinomas. TTF-1 is a marker of high sensitivity but insufficient specificity for pulmonary adenocarcinomas. A small fraction of TTF-1-positive gastrointestinal adenocarcinomas represents a pitfall mimicking enteric-type pulmonary adenocarcinoma. Combined analysis of TTF-1 and Napsin-A improves the specificity of pulmonary adenocarcinoma diagnosis.

**Supplementary Information:**

The online version contains supplementary material available at 10.1007/s00428-024-03926-1.

## Introduction

Thyroid transcription factor 1 (TTF-1) also termed homeobox protein Nkx-2.1 is a member of the NKX2 family of homeodomain transcription factors which promotes transcription in a tissue-specific manner and exerts a critical role in the development of the thyroid, the lung, and the brain. TTF-1 knock-out causes developmental disorders of these organs. In the thyroid gland, TTF-1 stimulates the expression of thyroid peroxidase, thyroglobulin, and thyrotropin receptors (summarized in [1]). In the lung, TTF-1 stimulates transcription of the surfactant proteins A to D and the Clara cell secretory protein [2, 3]. The molecular targets of TTF-1 in the brain are still unknown.

In normal adult extracranial tissues, TTF-1 expression is largely limited to specific cell types of the thyroid, the lung, and the pituitary gland [4–6]. Also in cancer, TTF-1 was shown to have high specificity for tumors derived from the lung and the thyroid. TTF-1 immunohistochemistry (IHC) is therefore routinely used in surgical pathology to support the difficult distinction of primary pulmonary adenocarcinomas which are often TTF-1 positive from malignant mesothelioma and metastatic adenocarcinoma to the lung which are usually TTF-1 negative [7]. Data on the rate of TTF-1 expression in cancer are heterogeneous, however (supplementary Fig. 1 and supplementary Table 1). Most studies evaluating TTF-1 by IHC have focused on pulmonary and thyroidal tumors and identified TTF-1 positivity in 42–100% of pulmonary adenocarcinomas [8–11], 15–93% of pulmonary small cell carcinomas [12–15], 0–50% of squamous cell carcinomas of the lung [12, 16–18], and 0–42% of malignant mesotheliomas [19–22] as well as in 88–100% of follicular [23, 24], 66–100% of papillary [23, 24], 50–100% of medullary [25, 26], and 5–100% of anaplastic cancers of the thyroid [27, 28]. Considerably, fewer authors analyzed TTF-1 expression in non-pulmonary/non-thyroidal cancers and reported—for example, TTF-1 positivity in 23–75% of endometrioid carcinomas of the ovary [29, 30], 5–80% of serous carcinomas of the endometrium [29, 30], 0–80% of Merkel cell carcinomas [31, 32], and 7–25% of endometrial clear cell carcinomas [29, 30]. Some of the divergent findings are likely due to the use of different antibodies, staining protocols, and criteria for defining TTF-1 positivity. For many tumor entities, immunochemical studies of TTF-1 expression in sizable cohorts are yet lacking.

To better understand the prevalence of TTF-1 expression in cancer, an extensive survey of TTF-1 immunostaining under standardized conditions in a broad range of tumor types is desirable. In this study, we therefore evaluated TTF-1 expression in more than 17,000 tumor tissue samples from 152 different tumor types and subtypes as well as 76 different non-neoplastic tissue types by IHC in a tissue microarray (TMA) format.

## Materials and methods

### Tissue microarrays (TMAs)

Our normal tissue TMA was composed of 8 samples from 8 different donors from 76 different normal tissue types (608 samples on one slide). The cancer TMAs contained a total of 17,772 primary tumors from 152 tumor types and subtypes. The composition of both normal and cancer TMAs is described in detail in the results section. All samples were from the archives of the Institute of Pathology, University Hospital of Hamburg, Germany, the Institute of Pathology, Clinical Center Osnabrueck, Germany, and Department of Pathology, Academic Hospital Fuerth, Germany. Tissues were fixed in 4% buffered formalin and then embedded in paraffin. The TMA manufacturing process was described earlier in detail [33, 34]. In brief, one tissue spot (diameter, 0.6 mm) per patient was used. The use of archived remnants of diagnostic tissues for manufacturing of TMAs and their analysis for research purposes as well as patient data analysis has been approved by local laws (HmbKHG, §12) and by the local ethics committee (Ethics commission Hamburg, WF-049/09). All work has been carried out in compliance with the Helsinki Declaration. Data on Napsin-A, cytokeratin 20 (CK20), DNA-binding protein SATB2 (SATB2), fatty acid-binding protein 1 (FABP1), Villin-1, progesterone receptor (PR), estrogen receptor (ER), and p53 were available for subsets of our tumors from previous studies [35–40] (data on ER and p53 are not published).

### Immunohistochemistry (IHC)

Freshly cut TMA sections were immunostained on one day and in one experiment. Slides were deparaffinized with xylol, rehydrated through a graded alcohol series and exposed to heat-induced antigen retrieval for 5 min in an autoclave at 121 °C in Dako Target Retrieval Solution, pH9 (Agilent Technologies, Santa Clara, CA, USA; #S2367). Endogenous peroxidase activity was blocked with Dako REAL Peroxidase-Blocking Solution (Agilent Technologies, Santa Clara, CA, USA; #S2023) for 10 min. Primary antibody specific for TTF-1 (recombinant rabbit monoclonal, MSVA-312R, MS Validated Antibodies, Hamburg, Germany; #5805-312R) was applied at 37 °C for 60 min at a dilution of 1:150. Bound antibody was then visualized using the Dako REAL EnVision Detection System Peroxidase/DAB + , Rabbit/Mouse kit (Agilent, Santa Clara, CA, USA; #K5007) according to the manufacturer’s directions. The sections were counterstained with hemalaun. For the purpose of antibody validation, the normal tissue TMA was also analyzed by the rabbit recombinant monoclonal anti-TTF1 antibody [EP1584Y] (Abcam, Cambridge, UK, #ab76013) at a dilution of 1:50 and an otherwise identical protocol. For tumor tissues, the percentage of positive neoplastic cells was estimated, and the staining intensity was semi-quantitatively recorded (0, 1 + , 2 + , 3 +). For statistical analyses, the staining results were categorized into four groups. Tumors without any staining were considered negative. Tumors with 1 + staining intensity in ≤ 70% of tumor cells and 2 + intensity in ≤ 30% of tumor cells were considered weakly positive. Tumors with 1 + staining intensity in > 70% of tumor cells, 2 + intensity in 31–70%, or 3 + intensity in ≤ 30% of tumor cells were considered moderately positive. Tumors with 2 + intensity in > 70% or 3 + intensity in > 30% of tumor cells were considered strongly positive.

Sensitivity and specificity of positive staining of TTF-1 alone, Napsin-A alone, or of both TTF-1 and Napsin-A for the distinction between adenocarcinomas of the lung and adenocarcinomas originating from other organs (including endometrium, ovary, breast, colon, stomach, esophagus, liver, bile ducts, pancreas, prostate, and adrenal gland), mesotheliomas, and renal cell carcinomas were calculated using the formulas “sensitivity = number of true positive / number of true positive + number of false negative” and “specificity = number of true negative / number of true negative + number of false positive.”

## Results

### Technical issues

A total of 15,564 (87.6%) of 17,772 tumor samples were interpretable in our TMA analysis. Non-interpretable samples demonstrated a lack of unequivocal tumor cells or an absence of entire tissue spots. A sufficient number of samples (≥ 4) of each normal tissue type was evaluable.

### TTF-1 in normal tissues

**Fig. 1:**
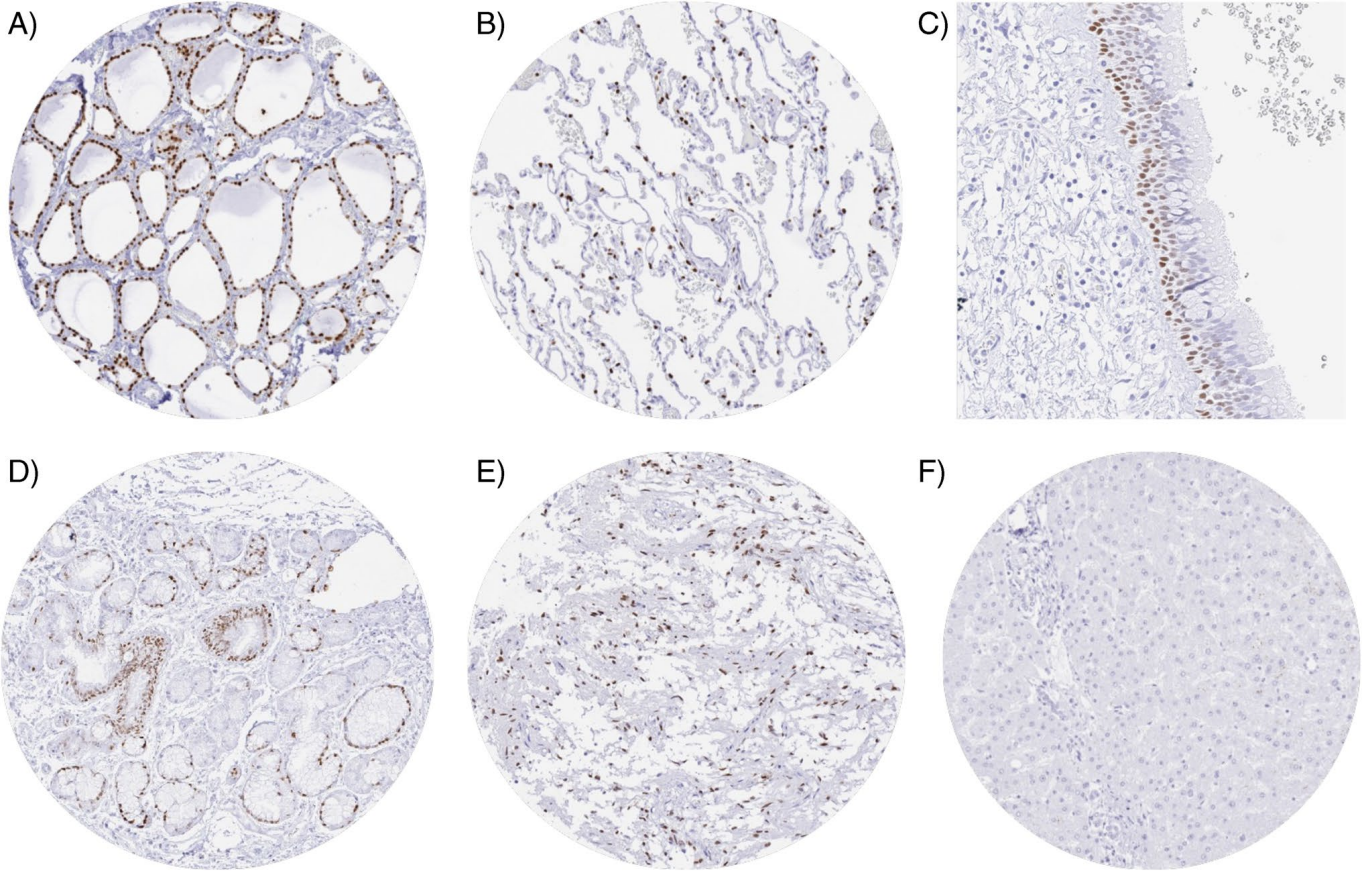
TTF-1 immunostaining in normal tissues. The panels show a moderate to strong nuclear TTF-1 staining of all epithelial cells of the thyroid (**A**), pneumocytes of the lung (**B**), basal cell layers of the respiratory epithelium of the bronchus (**C**), mucinous cells of bronchial glands (**D**), and in pituicytes of the neurohypophysis (**E**). TTF-1 staining is absent in all cells of the liver (**F**)

A strong nuclear TTF-1 staining was seen in all epithelial cells of the thyroid, pituicytes of the neurohypophysis, all pneumocytes of the lung, basal cell layers of respiratory epithelium of the bronchus, and in mucinous cells of bronchiolar glands. All these findings were obtained by using the recombinant rabbit monoclonal antibody MSVA-312R and the anti-TTF1 antibody [EP1584Y] and therefore considered to be specific. For MSVA-312R, TTF-1 staining was absent in skeletal muscle, heart muscle, smooth muscle, myometrium of the uterus, corpus spongiosum of the penis, ovarian stroma, fat, skin (including hair follicle and sebaceous glands), oral mucosa of the lip, oral cavity, surface epithelium of the tonsil, and transitional mucosa of the anal canal, ectocervix, squamous epithelium of the esophagus, urothelium of the renal pelvis and the urinary bladder, decidua, placenta, lymph node, spleen, thymus, tonsil, mucosa of the stomach, duodenum, ileum, appendix, colon, rectum and gall bladder, liver, pancreas, parotid gland, submandibular gland, sublingual gland, Brunner gland of the duodenum, cortex and medulla of the kidney, prostate, seminal vesicle, epididymis, breast, endocervix, endometrium, fallopian tube, adrenal gland, parathyroid gland, adenohypophysis, cerebellum, and the cerebrum. Representative images are shown in Fig. 1. By using the anti-TTF1 antibody clone [EP1584Y], additional cytoplasmic staining was seen in spermatogonia and spermatids of the testis (strong), smooth muscle (strong) as well as in several other normal tissues such as for example in renal tubule kidney (weaker). These stainings were considered antibody-specific cross-reactivities of [EP1584Y] (supplementary Fig. 2).

### TTF-1 in *cancer* tissues

**Fig. 2:**
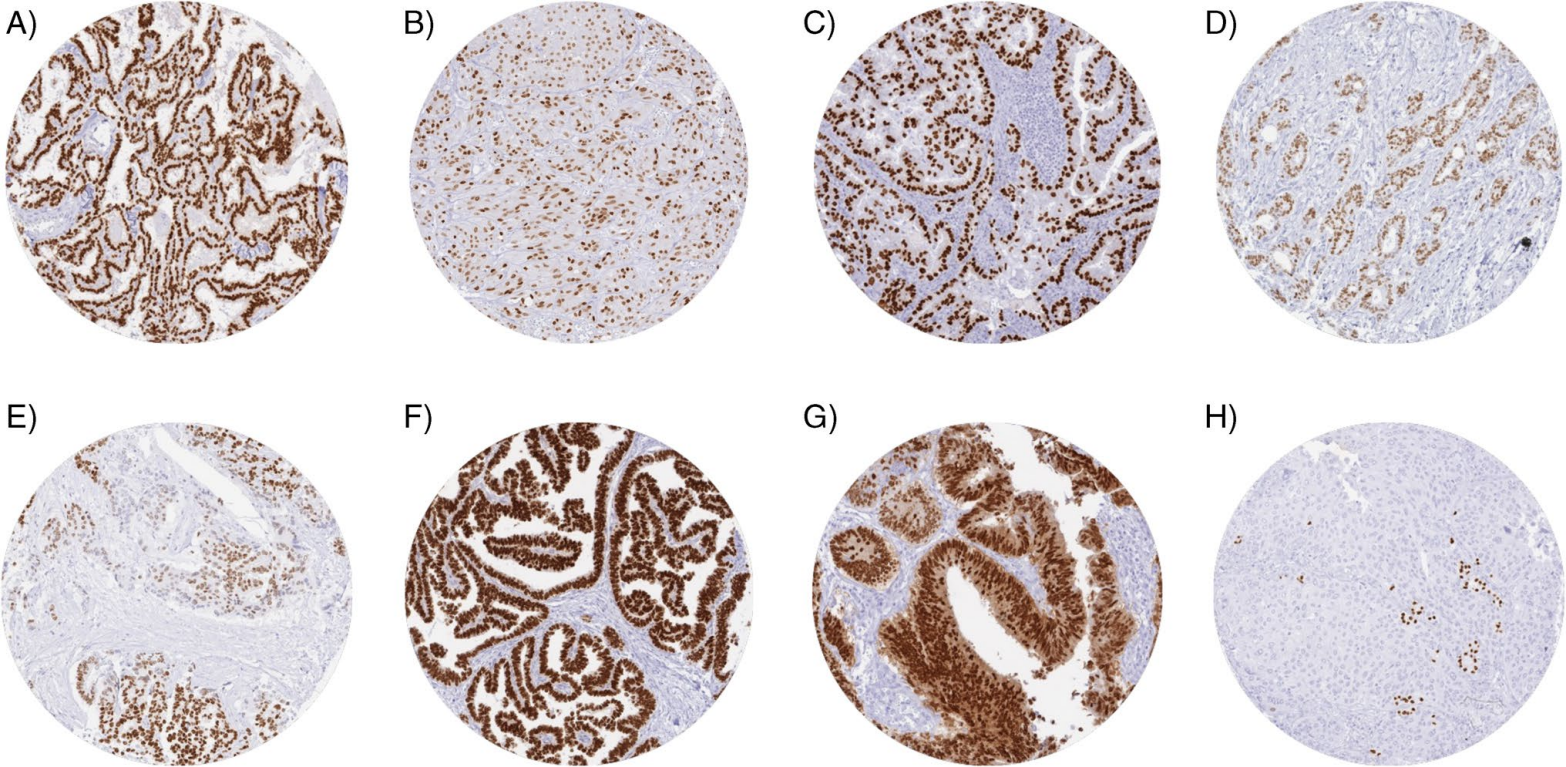
TTF-1 immunostaining in cancer tissues. The panels show a strong, nuclear TTF-1 positivity of all tumor cells in papillary (**A**) and medullary carcinoma (**B**) of the thyroid, pulmonary adenocarcinoma (**C**), adenocarcinoma (**D**), and neuroendocrine tumor of the pancreas (**E**), endometrioid carcinoma of the endometrium (**F**), and colorectal adenocarcinoma (**G**). In TTF-1 negative squamous cell carcinoma of the lung, TTF-1 staining is limited to retained normal pneumocytes (**H**)

TTF-1 immunostaining was detectable in 1389 (8.9%) of the 15,564 analyzable tumors, including 245 (1.6%) with weak, 139 (0.9%) with moderate, and 1005 (6.5%) with strong staining. Overall, 82 (53.9%) of 152 tumor categories showed detectable TTF-1 expression with 42 (27.6%) tumor categories including at least one case with strong positivity (Table 1). Representative images of TTF-1-positive tumors are shown in Fig. 2. The highest rate of TTF-1 positivity was found in various subtypes of thyroidal cancers (19.0–100%), adenocarcinomas of the lung (94.3%), neuroendocrine tumors (NET) of the lung (66.7%), small cell neuroendocrine carcinomas (NEC) of various organs of origin (71.4–80.0%), various categories of mesenchymal tumors (16.7–41.9%), and in thymomas (39.1%). TTF-1 expression in less than 15% of cases and often at lower intensity was also seen in various other cancer types such as gallbladder adenocarcinoma (14.3%), NEC of the ileum (14.3%), NET of the pancreas (5.4%), high-grade endometrial carcinoma (12.5%), urothelial carcinoma of the kidney pelvis (11.5%), adenocarcinoma of the prostate (up to 11.5%), carcinosarcoma of the uterus (9.3%), endometrial serous carcinoma (8.1%), tumors of the salivary gland (0.4–7.4%), pancreatic/ampullary adenocarcinoma (7.3%), squamous cell carcinomas from different organs (up to 6.8%, without lung), gastric adenocarcinoma (up to 5.9%), adenocarcinoma of the colon (5.2%), mucinous carcinoma of the ovary (4.7%), yolk sac tumor of the testis (4.7%), clear cell carcinoma of the ovary (4.3%), endometrioid endometrial carcinoma (4.1%), T-cell non-Hodgkin’s lymphoma (4.0%), and in cholangiocarcinoma (3.5%). The prevalence of TTF-1 in different categories of neuroendocrine neoplasms is also shown separately in Table 2. Four out of nine TTF-1 positive endometrial carcinomas with data on ER, PR, and p53 from previous studies ([40] data on ER and p53 are not published) demonstrated a wildtype pattern of p53 and absence of ER and PR immunostaining, in combination with the morphological features in line with the “mesonephric-like endometrial carcinoma” subtype.

**Table 1:** TTF-1 immunostaining in human tumors

Tumor category	Tumor entity	TTF-1 immunostaining					
		TMA (*n*)	Analyzable (*n*)	Negative (%)	Weak (%)	Moderate (%)	Strong (%)
Tumors of the skin	Pilomatricoma	35	13	100.0	0.0	0.0	0.0
	Basal cell carcinoma of the skin	89	79	98.7	1.3	0.0	0.0
	Benign nevus	29	22	100.0	0.0	0.0	0.0
	Squamous cell carcinoma of the skin	145	130	96.9	2.3	0.8	0.0
	Malignant melanoma	65	57	100.0	0.0	0.0	0.0
	Malignant melanoma lymph node metastasis	86	85	98.8	0.0	1.2	0.0
	Merkel cell carcinoma	48	28	92.9	0.0	7.1	0.0
**Tumors of the head and neck**	Squamous cell carcinoma of the larynx	109	100	97.0	3.0	0.0	0.0
	Squamous cell carcinoma of the pharynx	60	59	93.2	5.1	1.7	0.0
	Oral squamous cell carcinoma (floor of the mouth)	130	126	97.6	2.4	0.0	0.0
	Pleomorphic adenoma of the parotid gland	50	36	100.0	0.0	0.0	0.0
	Warthin tumor of the parotid gland	104	89	100.0	0.0	0.0	0.0
	Adenocarcinoma, NOS (Papillary Cystadenocarcinoma)	14	11	100.0	0.0	0.0	0.0
	Salivary duct carcinoma	15	9	100.0	0.0	0.0	0.0
	Acinic cell carcinoma of the salivary gland	181	103	99.0	0.0	1.0	0.0
	Adenocarcinoma NOS of the salivary gland	109	54	92.6	3.7	1.9	1.9
	Adenoid cystic carcinoma of the salivary gland	180	64	100.0	0.0	0.0	0.0
	Basal cell adenocarcinoma of the salivary gland	25	21	100.0	0.0	0.0	0.0
	Basal cell adenoma of the salivary gland	101	58	100.0	0.0	0.0	0.0
	Epithelial-myoepithelial carcinoma of the salivary gland	53	43	100.0	0.0	0.0	0.0
	Mucoepidermoid carcinoma of the salivary gland	343	261	99.6	0.0	0.0	0.4
	Myoepithelial carcinoma of the salivary gland	21	15	93.3	0.0	6.7	0.0
	Myoepithelioma of the salivary gland	11	9	100.0	0.0	0.0	0.0
	Oncocytic carcinoma of the salivary gland	12	6	100.0	0.0	0.0	0.0
	Polymorphous adenocarcinoma, low grade, of the salivary gland	41	21	100.0	0.0	0.0	0.0
	Pleomorphic adenoma of the salivary gland	53	31	100.0	0.0	0.0	0.0
**Tumors of the lung, pleura and thymus**	Adenocarcinoma of the lung	196	175	5.7	3.4	3.4	87.4
	Squamous cell carcinoma of the lung	80	66	84.8	1.5	3.0	10.6
	Small cell carcinoma of the lung	16	5	20.0	0.0	0.0	80.0
	Mesothelioma, epithelioid	40	34	100.0	0.0	0.0	0.0
	Mesothelioma, biphasic	77	43	100.0	0.0	0.0	0.0
	Thymoma	29	23	60.9	26.1	4.3	8.7
	Lung, neuroendocrine tumor (NET)	29	24	33.3	0.0	4.2	62.5
**Tumors of the female genital tract**	Squamous cell carcinoma of the vagina	78	68	97.1	1.5	1.5	0.0
	Squamous cell carcinoma of the vulva	157	148	98.0	1.4	0.7	0.0
	Squamous cell carcinoma of the cervix	136	131	99.2	0.0	0.8	0.0
	Adenocarcinoma of the cervix	23	23	100.0	0.0	0.0	0.0
	Endometrioid endometrial carcinoma	338	295	95.9	1.4	1.4	1.4
	Endometrial serous carcinoma	86	74	91.9	5.4	0.0	2.7
	Carcinosarcoma of the uterus	57	54	90.7	5.6	1.9	1.9
	Endometrial carcinoma, high grade, G3	13	8	87.5	12.5	0.0	0.0
	Endometrial clear cell carcinoma	9	6	100.0	0.0	0.0	0.0
	Endometrioid carcinoma of the ovary	130	118	97.5	0.8	0.0	1.7
	Serous carcinoma of the ovary	580	504	97.4	1.4	1.2	0.0
	Mucinous carcinoma of the ovary	101	86	95.3	2.3	0.0	2.3
	Clear cell carcinoma of the ovary	51	46	95.7	0.0	0.0	4.3
	Carcinosarcoma of the ovary	47	47	100.0	0.0	0.0	0.0
	Granulosa cell tumor of the ovary	44	44	100.0	0.0	0.0	0.0
	Leydig cell tumor of the ovary	4	4	100.0	0.0	0.0	0.0
	Sertoli cell tumor of the ovary	1	1	100.0	0.0	0.0	0.0
	Sertoli Leydig cell tumor of the ovary	3	3	100.0	0.0	0.0	0.0
	Steroid cell tumor of the ovary	3	3	100.0	0.0	0.0	0.0
	Brenner tumor	41	41	100.0	0.0	0.0	0.0
**Tumors of the breast**	Invasive breast carcinoma of no special type	499	492	99.2	0.4	0.2	0.2
	Lobular carcinoma of the breast	192	189	98.9	0.5	0.0	0.5
	Medullary carcinoma of the breast	23	23	100.0	0.0	0.0	0.0
	Tubular carcinoma of the breast	20	17	100.0	0.0	0.0	0.0
	Mucinous carcinoma of the breast	29	28	100.0	0.0	0.0	0.0
	Phyllodes tumor of the breast	50	46	100.0	0.0	0.0	0.0
**Tumors of the digestive system**	Adenomatous polyp, low-grade dysplasia	50	50	98.0	2.0	0.0	0.0
	Adenomatous polyp, high-grade dysplasia	50	49	89.8	8.2	2.0	0.0
	Adenocarcinoma of the colon	2483	2290	94.8	2.4	0.9	1.9
	Gastric adenocarcinoma, diffuse type	215	167	100.0	0.0	0.0	0.0
	Gastric adenocarcinoma, intestinal type	215	188	94.1	4.3	1.1	0.5
	Gastric adenocarcinoma, mixed type	62	60	95.0	3.3	0.0	1.7
	Adenocarcinoma of the esophagus	83	65	96.9	1.5	0.0	1.5
	Squamous cell carcinoma of the esophagus	76	55	100.0	0.0	0.0	0.0
	Squamous cell carcinoma of the anal canal	91	86	97.7	2.3	0.0	0.0
	Cholangiocarcinoma	121	114	96.5	0.9	1.8	0.9
	Gallbladder adenocarcinoma	51	49	85.7	10.2	0.0	4.1
	Gallbladder Klatskin tumor	42	39	94.9	5.1	0.0	0.0
	Hepatocellular carcinoma	312	307	98.7	1.0	0.3	0.0
	Ductal adenocarcinoma of the pancreas	659	624	98.2	1.0	0.5	0.3
	Pancreatic/Ampullary adenocarcinoma	98	96	92.7	5.2	2.1	0.0
	Acinar cell carcinoma of the pancreas	18	18	100.0	0.0	0.0	0.0
	Gastrointestinal stromal tumor (GIST)	62	58	100.0	0.0	0.0	0.0
	Appendix, neuroendocrine tumor (NET)	25	16	100.0	0.0	0.0	0.0
	Colorectal, neuroendocrine tumor (NET)	12	11	100.0	0.0	0.0	0.0
	Ileum, neuroendocrine tumor (NET)	53	51	100.0	0.0	0.0	0.0
	Pancreas, neuroendocrine tumor (NET)	101	93	94.6	0.0	1.1	4.3
	Colorectal, neuroendocrine carcinoma (NEC)	14	12	91.7	0.0	8.3	0.0
	Ileum, neuroendocrine carcinoma (NEC)	8	7	85.7	0.0	14.3	0.0
	Gallbladder, neuroendocrine carcinoma (NEC)	4	4	0.0	100.0	0.0	0.0
	Pancreas, neuroendocrine carcinoma (NEC)	14	14	85.7	7.1	0.0	7.1
**Tumors of the urinary system**	Non-invasive papillary urothelial carcinoma, pTa G2 low grade	177	158	93.0	5.1	1.9	0.0
	Non-invasive papillary urothelial carcinoma, pTa G2 high grade	141	117	89.7	4.3	5.1	0.9
	Non-invasive papillary urothelial carcinoma, pTa G3	219	126	97.6	0.0	2.4	0.0
	Urothelial carcinoma, pT2-4 G3	735	616	97.1	1.3	0.5	1.1
	Squamous cell carcinoma of the bladder	22	22	100.0	0.0	0.0	0.0
	Small cell neuroendocrine carcinoma of the bladder	23	15	26.7	0.0	6.7	66.7
	Sarcomatoid urothelial carcinoma	25	23	95.7	4.3	0.0	0.0
	Urothelial carcinoma of the kidney pelvis	62	61	88.5	6.6	3.3	1.6
	Clear cell renal cell carcinoma	1286	1224	99.9	0.0	0.1	0.0
	Papillary renal cell carcinoma	368	327	99.1	0.9	0.0	0.0
	Clear cell (tubulo) papillary renal cell carcinoma	26	23	100.0	0.0	0.0	0.0
	Chromophobe renal cell carcinoma	170	151	99.3	0.7	0.0	0.0
	Oncocytoma of the kidney	257	228	100.0	0.0	0.0	0.0
**Tumors of the male genital organs**	Adenocarcinoma of the prostate, Gleason 3 + 3	83	80	100.0	0.0	0.0	0.0
	Adenocarcinoma of the prostate, Gleason 4 + 4	80	71	94.4	4.2	1.4	0.0
	Adenocarcinoma of the prostate, Gleason 5 + 5	85	79	91.1	8.9	0.0	0.0
	Adenocarcinoma of the prostate (recurrence)	258	218	88.5	9.2	1.4	0.9
	Small cell neuroendocrine carcinoma of the prostate	19	7	28.6	0.0	0.0	71.4
	Seminoma	682	575	100.0	0.0	0.0	0.0
	Embryonal carcinoma of the testis	54	37	100.0	0.0	0.0	0.0
	Leydig cell tumor of the testis	31	31	100.0	0.0	0.0	0.0
	Sertoli cell tumor of the testis	2	2	100.0	0.0	0.0	0.0
	Sex cord stromal tumor of the testis	1	1	100.0	0.0	0.0	0.0
	Spermatocytic tumor of the testis	1	1	100.0	0.0	0.0	0.0
	Yolk sac tumor	53	43	95.3	2.3	2.3	0.0
	Teratoma	53	39	94.9	0.0	2.6	2.6
	Squamous cell carcinoma of the penis	92	90	96.7	2.2	1.1	0.0
**Tumors of endocrine organs**	Adenoma of the thyroid gland	113	107	0.0	1.9	0.9	97.2
	Papillary thyroid carcinoma	391	373	0.8	0.0	0.8	98.4
	Follicular thyroid carcinoma	154	145	0.0	0.0	2.1	97.9
	Medullary thyroid carcinoma	111	96	0.0	1.0	18.8	80.2
	Parathyroid gland adenoma	43	42	100.0	0.0	0.0	0.0
	Anaplastic thyroid carcinoma	45	42	81.0	2.4	2.4	14.3
	Adrenal cortical adenoma	50	38	100.0	0.0	0.0	0.0
	Adrenal cortical carcinoma	28	28	100.0	0.0	0.0	0.0
	Pheochromocytoma	50	50	100.0	0.0	0.0	0.0
**Tumors of hematopoietic and lymphoid tissues**	Hodgkin’s lymphoma	103	89	98.9	1.1	0.0	0.0
	Small lymphocytic lymphoma, B-cell type (B-SLL/B-CLL)	50	50	100.0	0.0	0.0	0.0
	Diffuse large B-cell lymphoma (DLBCL)	113	113	98.2	1.8	0.0	0.0
	Follicular lymphoma	88	88	100.0	0.0	0.0	0.0
	T-cell non-Hodgkin’s lymphoma	25	25	96.0	0.0	0.0	4.0
	Mantle cell lymphoma	18	18	100.0	0.0	0.0	0.0
	Marginal zone lymphoma	16	16	100.0	0.0	0.0	0.0
	Diffuse large B-cell lymphoma (DLBCL) in the testis	16	16	100.0	0.0	0.0	0.0
	Burkitt lymphoma	5	5	100.0	0.0	0.0	0.0
**Tumors of soft tissue and bone**	Tendosynovial giant cell tumor	45	16	100.0	0.0	0.0	0.0
	Granular cell tumor	53	29	100.0	0.0	0.0	0.0
	Leiomyoma	50	44	100.0	0.0	0.0	0.0
	Leiomyosarcoma	94	86	98.8	1.2	0.0	0.0
	Liposarcoma	145	105	99.0	0.0	1.0	0.0
	Malignant peripheral nerve sheath tumor (MPNST)	15	14	78.6	7.1	14.3	0.0
	Myofibrosarcoma	26	26	100.0	0.0	0.0	0.0
	Angiosarcoma	74	50	100.0	0.0	0.0	0.0
	Angiomyolipoma	91	89	98.9	1.1	0.0	0.0
	Dermatofibrosarcoma protuberans	21	16	100.0	0.0	0.0	0.0
	Ganglioneuroma	14	12	100.0	0.0	0.0	0.0
	Kaposi sarcoma	8	5	100.0	0.0	0.0	0.0
	Neurofibroma	117	117	99.1	0.0	0.0	0.9
	Sarcoma, not otherwise specified (NOS)	74	71	94.4	2.8	1.4	1.4
	Paraganglioma	41	40	100.0	0.0	0.0	0.0
	Ewing sarcoma	23	18	83.3	11.1	0.0	5.6
	Rhabdomyosarcoma	7	7	71.4	0.0	14.3	14.3
	Schwannoma	122	117	58.1	10.3	12.0	19.7
	Synovial sarcoma	12	9	100.0	0.0	0.0	0.0
	Osteosarcoma	44	27	100.0	0.0	0.0	0.0
	Chondrosarcoma	40	16	100.0	0.0	0.0	0.0
	Rhabdoid tumor	5	5	80.0	20.0	0.0	0.0
	Solitary fibrous tumor	17	17	100.0	0.0	0.0	0.0

**Table 2:** Prevalence of TTF-1 in neuroendocrine neoplasms

Tumor entity	TTF-1 immunostaining			
	*n*	Weak (%)	Moderate (%)	Strong (%)
Gallbladder, neuroendocrine carcinoma (NEC)	4	100.0	0.0	0.0
Lung, neuroendocrine tumor (NET)	24	0.0	4.2	62.5
Ileum, neuroendocrine carcinoma (NEC)	7	0.0	14.3	0.0
Pancreas, neuroendocrine carcinoma (NEC)	14	7.1	0.0	7.1
Colorectal, neuroendocrine carcinoma (NEC)	12	0.0	8.3	0.0
Pancreas, neuroendocrine tumor (NET)	93	0.0	1.1	4.3
Appendix, neuroendocrine tumor (NET)	16	0.0	0.0	0.0
Colorectal, neuroendocrine tumor (NET)	11	0.0	0.0	0.0
Ileum, neuroendocrine tumor (NET)	51	0.0	0.0	0.0

### TTF-1 vs. Napsin-A and markers for enteric differentiation

The comparative analysis of TTF-1 vs. Napsin-A for their sensitivity and specificity for the distinction of pulmonary adenocarcinomas from other tumors (except thyroidal cancers) in a subset of 4567 cancers with data for both TTF-1 and Napsin-A revealed a higher sensitivity for TTF-1 (94.1%) while the specificity was higher for Napsin-A (97.8%, Table 3). The combination of TTF-1 and Napsin-A positivity resulted in a specificity of 99.1% for pulmonary adenocarcinoma (Table 3). The relationship between the expression of TTF-1, Napsin-A, and several enteric markers (CK20, SATB2, FABP1, Villin-1) in pulmonary, colorectal, pancreatic, and gastric adenocarcinomas is given in Table 4. This analysis revealed an expression of at least one enteric marker in 22.1% of 68 TTF-1 positive pulmonary adenocarcinomas while TTF-1 positivity was also seen in 66 colorectal, 9 pancreatic, and 8 gastric adenocarcinomas. Of these, 4 pancreatic adenocarcinomas were negative for all 4 enteric markers. Of note, Napsin-A was negative in all TTF-1-positive gastrointestinal adenocarcinomas.

**Table 3:** Comparison of the sensitivity and specificity of TTF-1 and Napsin-A (alone or together) for the distinction between lung adenocarcinomas and adenocarcinomas of other origin

	Sensitivity	Specificity
TTF-1 positive	0.941	0.861
Napsin-A positive	0.874	0.978
TTF-1 and Napsin-A positive	0.849	0.991

**Table 4:** Expression of typical enteric markers, including cytokeratin 20 (CK20), DNA-binding protein SATB2 (SATB2), fatty acid-binding protein 1 (FABP1), and Villin-1 in pulmonary, colorectal, pancreatic, and gastric adenocarcinomas

	*n*	Enteric markers					Number of positive enteric markers			
		Napsin-A positive (%)	CK20 positive (%)	SATB2 positive (%)	FABP1 positive (%)	Villin-1 positive (%)	4 positive (%)	3 positive (%)	2 positive (%)	1 positive (%)
Colon Carcinoma (TTF-1 positive)	66	0	100	95.5	84.8	100	84.8	10.6	4.5	0
Pancreatic carcinoma (TTF-1 positive)	9	0	22.2	22.2	0	22.2	0	0	11.1	44.4
Gastric carcinoma (TTF-1 positive)	8	0	50	62.5	25	100	25	12.5	37.5	25
Lung adenocarcinoma (TTF-1 positive)	68	89.7	2.9	10.3	0	16.2	0	0	7.4	14.7
Lung adenocarcinoma (TTF-1 negative)	4	50	0	0	0	0	0	0	0	0
Lung adenocarcinoma with enteric features	15	86.7								

## Discussion

TTF-1 IHC is widely used by pathologists for the distinction of primary pulmonary adenocarcinoma—typically TTF-1 positive—from pulmonary squamous cell carcinoma, metastatic adenocarcinoma to the lung, and pleural mesothelioma which are TTF-1 negative in most cases [7, 13, 41]. TTF-1 IHC has also been suggested to assist in the distinction of pulmonary from non-pulmonary neuroendocrine neoplasms [42], the distinction of Merkel cell carcinoma of the skin from cutaneous metastases of pulmonary small cell carcinoma [43], and as a marker for thyroidal carcinomas of all types including medullary carcinoma [44]. The results from our successful analysis of more than 15,000 cancers from 152 tumor entities confirm the suggested utility of TTF-1 IHC for these applications but also highlight important pitfalls and emphasize the significance of combining TTF-1 analysis with Napsin-A and other immunohistochemical markers.

Because the lung is a common site of metastases, the distinction of primary lung adenocarcinoma from metastatic adenocarcinoma is a frequent diagnostic problem with substantial therapeutic implications [45]. Although our data show a very high sensitivity (94.1%) of TTF-1 IHC for lung adenocarcinomas, it is conspicuous that its specificity (86%) is not optimal for a safe separation of pulmonary adenocarcinomas from morphologically similar metastatic cancers. The suboptimal specificity is due to small but significant fractions of TTF-1 positive cases in many common tumor entities such as adenocarcinomas of the colorectum, stomach, pancreas, prostate, ovary, and uterus, as well as urothelial carcinomas. All these tumors do often metastasize to the lung (summarized in [46]). Napsin-A is another commonly used marker for the distinction of pulmonary adenocarcinoma which was analyzed in our tumor cohort earlier [35]. While the sensitivity of Napsin-A (87.4%) is lower than for TTF-1, Napsin-A positivity is markedly more specific (97.8%) for pulmonary adenocarcinoma. In a combined analysis of both markers, our data suggest a good sensitivity (84.9%) and a close to perfect specificity (99.1%) for the combination TTF-1 + /Napsin-A + to distinguish pulmonary adenocarcinoma from its differential diagnoses.

Several authors had suggested that occasional TTF-1-positive gastrointestinal adenocarcinomas would be distinguishable from pulmonary adenocarcinomas by their additional expression of “gastrointestinal markers” [47–49]. That a large fraction of our TTF-1 positive gastric, pancreatic, and colorectal carcinomas was indeed positive for at least one of the markers CK20, Villin-1, SATB2, and FABP1 is supportive of this concept, but expression of at least one of these markers also occurred in 22% of our TTF-1 positive pulmonary adenocarcinomas. Such “enteric type” pulmonary adenocarcinomas have been described to make up for 3–28% of pulmonary adenocarcinomas [50–52]. Based on our set of data, these tumors represent an important pitfall which can often (> 80%) be distinguished from gastrointestinal tumors by their Napsin-A positivity, a feature that was not seen in any of our gastrointestinal neoplasms.

Our data also enabled an assessment of the previously proposed utility of TTF-1 IHC for the distinction of pulmonary from extrapulmonary neuroendocrine neoplasms and demonstrated that this separation can best be made in highly differentiated tumors. The 67% TTF-1 positivity of pulmonary NETs (carcinoids) is close to the average of 47% TTF-1 positive lung carcinoids described in previous studies although the reported positivity rates in these tumors ranged from 0 to 86% [16, 53]. Pancreatic NETs may show the second-highest rate of TTF-1 positivity among NETs. Our rate of TTF-1 positivity in 5.4% of 93 pancreatic NETs is close to the 7% of 44 described by Tseng et al. [42] while Koo et al. [54] had not found TTF-1 expression in a series of 33 cases. The complete absence of TTF-1 staining in NETs from the ileum (*n* = 51), appendix (*n* = 16), and colorectum (*n* = 11) is consistent with reports describing a lack of TTF-1 positivity in 17 and 23 NETs of the ileum [42, 54], 6 NETs of the appendix [42], and 14 and 23 NETs of the rectum [42, 54]. It is of note that the frequency of TTF-1 positivity in extrapulmonary neuroendocrine neoplasms increases with tumor dedifferentiation (summarized in [55]). Accordingly, at least a few TTF-1 positive cases were seen in NECs from almost every site of origin and an even higher TTF-1 positivity rate occurred in small cell NECs from extra-thoracic sites of origin in this study. Similar findings had been described by other authors [42]. It is generally accepted that TTF-1 immunostaining cannot be used to distinguish the site of origin in case of small cell NEC (summarized in [55]). That two (7%) of our 28 Merkel cell carcinomas showed a moderate TTF-1 positivity is consistent with the sum of earlier data describing TTF-1 positivity in 0% of 12 [56], 0% of 20 [31], 8% of 52 [57], 11% of 103 [58], and 80% of 5 [32] Merkel cell carcinomas. These findings demonstrate that TTF-1-positive small cell tumors of the skin are not always of metastatic origin.

The comprehensive evaluation of more than 15,000 tumors identified several further tumor entities with frequent TTF-1 positivity some of which were previously underrecognized. These include several mesenchymal neoplasms. Of these, malignant peripheral nerve sheath tumors (MPNST) can for example occur in the thyroid and may mimic anaplastic thyroid cancer [59]. TTF-1-positive Ewing sarcoma may erroneously give rise to the diagnosis of a bone metastasis derived from a small cell NEC of the lung or of another site of origin. TTF-1 positivity in thymoma, T-cell lymphoma, and large cell B-cell lymphoma may also cause diagnostic confusion if seen in thoracic or lympho-nodal tumor masses.

Given the large scale of our study, emphasis was placed on a thorough validation of our assay. The International Working Group for Antibody Validation (IWGAV) has proposed that antibody validation for IHC on formalin-fixed tissues should include either a comparison of the findings obtained by two different independent antibodies or a comparison with expression data obtained by another independent method [60]. RNA data obtained in three independent RNA screening studies [61–64] had identified TTF-1 RNA only in the thyroid, lung, pituitary gland, and brain. That TTF-1 staining by MSVA-312R was also restricted to thyroid, neurohypophysis, and pulmonary tissues, and that all cell types that were TTF-1 positive by MSVA-312R were also labeled by [EP1584Y] constitutes a strong validation of our assay. It is of note that our broad panel of 76 different normal tissues for antibody validation results in a high likelihood for detecting undesired cross-reactivities because virtually all proteins occurring in normal cells of adult humans are subjected to the validation experiment. The cytoplasmic TTF-1 staining seen in various tissues by [EP1584Y] but not by MSVA-312R represents an antibody-specific cross-reactivity of [EP1584Y] which was identified by our resource-intensive validation procedure.

## Conclusion

Our data reveal that TTF-1 is a marker of high sensitivity but insufficient specificity for the distinction of pulmonary adenocarcinomas. A small fraction of TTF-1-positive gastrointestinal adenocarcinomas represents a significant pitfall mimicking enteric-type pulmonary adenocarcinoma. The combined analysis of TTF-1 and Napsin-A significantly improves the specificity of pulmonary adenocarcinoma diagnosis.

## Supplementary Information

Below is the link to the electronic supplementary material.

Supplementary Fig. 1 Comparison with previous TTF-1 literature. An „X “ indicates the fraction of TTF-1 positive cancers in the present study, dots indicate the reported frequencies from the literature for comparison: red dots mark studies with ≤ 10 analyzed tumors, yellow dots mark studies with 11 to 25 analyzed tumors, and green dots mark studies with > 25 analyzed tumors. References are found in supplementary Table 1. (PDF 39 KB)

Supplementary Supplementary Fig. 2 Immunohistochemistry (IHC) validation by comparison of two antibodies. The panels show a confirmation of immunostaining results obtained by MSVA-312R. Using MSVA-312R, the panels show a very intense nuclear TTF-1 positivity of follicular cells of the thyroid (A) and of pneumocytes of the lung (B), a moderate to strong staining of respiratory epithelial cells (C), mucinous bronchial glands (D), and pituicytes of the neurohypophysis (E) while TTF-1 staining is absent in testis (F), kidney (G), and the ileum (H). Using clone [EP1584Y], a comparable nuclear staining is seen in the thyroid (I), lung (K), respiratory epithelium (L), mucinous bronchial glands (M), and the hypophysis (N) although an additional moderate to strong cytoplasmic staining occurs in respiratory epithelial cells (L) spermatogonia and spermatides of the testis (O), renal tubular cells (P), and the muscularis mucosae of the ileum (Q). The images A-H and I-Q are from consecutive tissue sections. (PDF 1151 KB)

Supplementary Table 1 Previous TTF-1 immunohistochemistry studies. (DOCX 28 KB)

Supplementary file4 (DOCX 13 KB)

## Data Availability

Raw data are available upon reasonable request. All data relevant to the study are included in the article.
