# Supplementary figures and images for: TTF-1 is a highly sensitive but not fully specific marker for pulmonary and thyroidal cancer: a tissue microarray study evaluating more than 17,000 tumors from 152 different tumor entities
Source: Virchows Arch. 2024 Oct 8;485(5):815–28. doi: 10.1007/s00428-024-03926-1 (PMC11564378; doi:10.1007/s00428-024-03926-1)

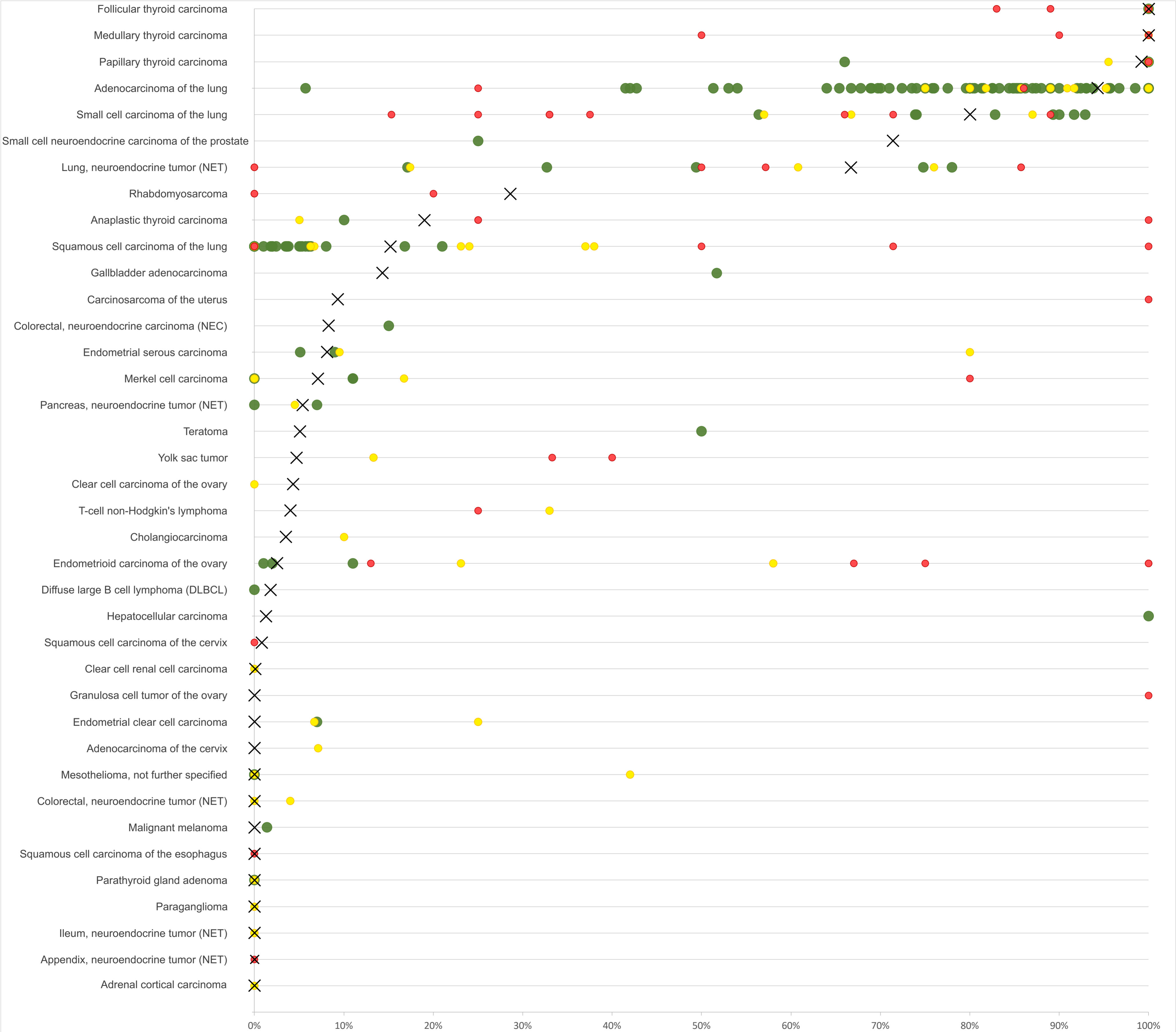

Supplement: Supplementary file 1 — Supplementary Fig. 1 Comparison with previous TTF-1 literature. An „X “ indicates the fraction of TTF-1 positive cancers in the present study, dots indicate the reported frequencies from the literature for comparison: red dots mark studies with ≤ 10 analyzed tumors, yellow dots mark studies with 11 to 25 analyzed tumors, and green dots mark studies with > 25 analyzed tumors. References are found in supplementary Table 1. (PDF 39 KB) [file 428_2024_3926_MOESM1_ESM.pdf]

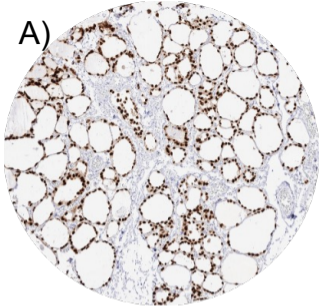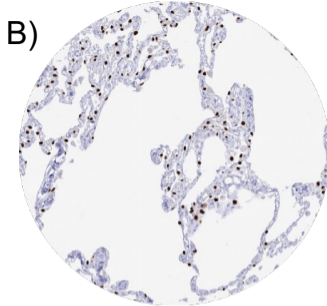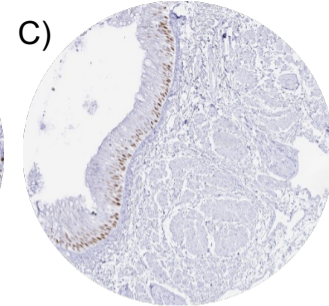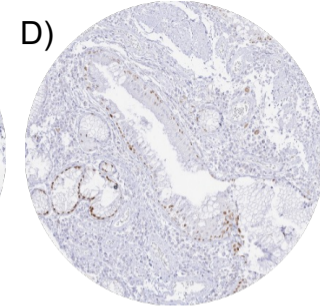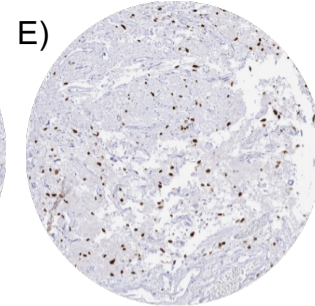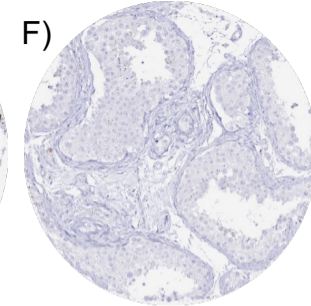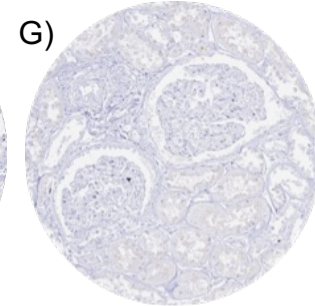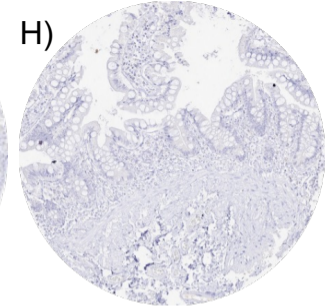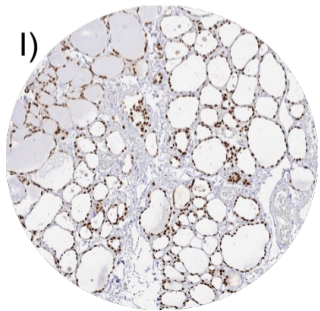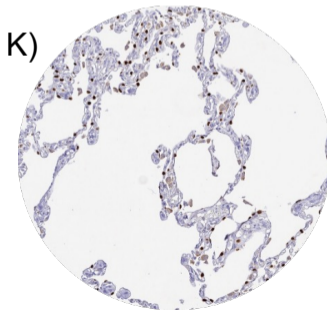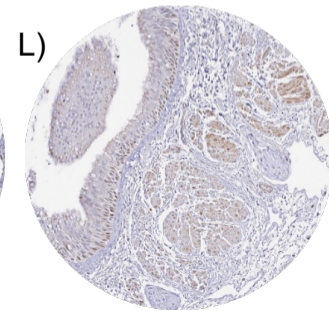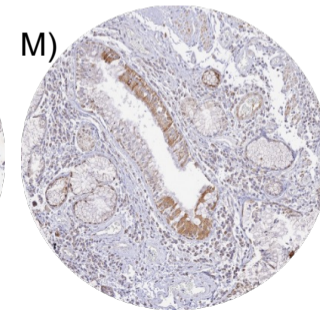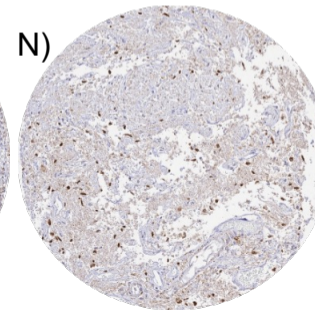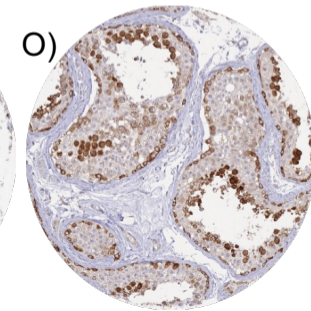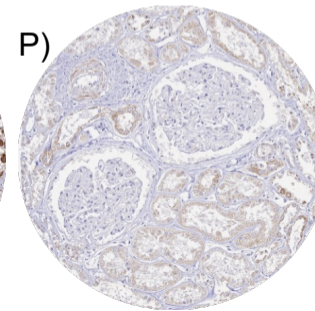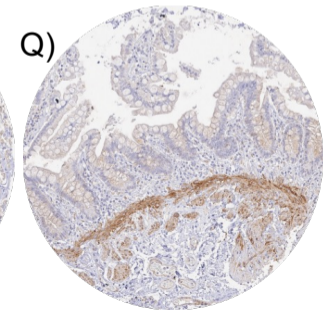

Supplement: Supplementary file 2 — Supplementary Supplementary Fig. 2 Immunohistochemistry (IHC) validation by comparison of two antibodies. The panels show a confirmation of immunostaining results obtained by MSVA-312R. Using MSVA-312R, the panels show a very intense nuclear TTF-1 positivity of follicular cells of the thyroid (A) and of pneumocytes of the lung (B), a moderate to strong staining of respiratory epithelial cells (C), mucinous bronchial glands (D), and pituicytes of the neurohypophysis (E) while TTF-1 staining is absent in testis (F), kidney (G), and the ileum (H). Using clone [EP1584Y], a comparable nuclear staining is seen in the thyroid (I), lung (K), respiratory epithelium (L), mucinous bronchial glands (M), and the hypophysis (N) although an additional moderate to strong cytoplasmic staining occurs in respiratory epithelial cells (L) spermatogonia and spermatides of the testis (O), renal tubular cells (P), and the muscularis mucosae of the ileum (Q). The images A-H and I-Q are from consecutive tissue sections. (PDF 1151 KB) [file 428_2024_3926_MOESM2_ESM.pdf]
