# Supplementary material for: TTF-1 is a highly sensitive but not fully specific marker for pulmonary and thyroidal cancer: a tissue microarray study evaluating more than 17,000 tumors from 152 different tumor entities
Source: Virchows Arch. 2024 Oct 8;485(5):815–28. doi: 10.1007/s00428-024-03926-1 (PMC11564378; doi:10.1007/s00428-024-03926-1)
Supplement: Supplementary file 3 — Supplementary Table 1 Previous TTF-1 immunohistochemistry studies. (DOCX 28 KB) [file 428_2024_3926_MOESM3_ESM.docx]

|  |  |  | **TTF-1 immunostaining** | | | | |
| --- | --- | --- | --- | --- | --- | --- | --- |
| **Tumor category** | **Tumor entity** | on TMA (n) | analyzable (n) | negative (%) | weak (%) | moderate (%) | strong (%) |
| **Tumors of the skin** | Pilomatricoma | 35 | 13 | 100.0 | 0.0 | 0.0 | 0.0 |
|  | Basal cell carcinoma of the skin | 89 | 79 | 98.7 | 1.3 | 0.0 | 0.0 |
|  | Benign nevus | 29 | 22 | 100.0 | 0.0 | 0.0 | 0.0 |
|  | Squamous cell carcinoma of the skin | 145 | 130 | 96.9 | 2.3 | 0.8 | 0.0 |
|  | Malignant melanoma | 65 | 57 | 100.0 | 0.0 | 0.0 | 0.0 |
|  | Malignant melanoma lymph node metastasis | 86 | 85 | 98.8 | 0.0 | 1.2 | 0.0 |
|  | Merkel cell carcinoma | 48 | 28 | 92.9 | 0.0 | 7.1 | 0.0 |
| **Tumors of the head and neck** | Squamous cell carcinoma of the larynx | 109 | 100 | 97.0 | 3.0 | 0.0 | 0.0 |
|  | Squamous cell carcinoma of the pharynx | 60 | 59 | 93.2 | 5.1 | 1.7 | 0.0 |
|  | Oral squamous cell carcinoma (floor of the mouth) | 130 | 126 | 97.6 | 2.4 | 0.0 | 0.0 |
|  | Pleomorphic adenoma of the parotid gland | 50 | 36 | 100.0 | 0.0 | 0.0 | 0.0 |
|  | Warthin tumor of the parotid gland | 104 | 89 | 100.0 | 0.0 | 0.0 | 0.0 |
|  | Adenocarcinoma, NOS (Papillary Cystadenocarcinoma) | 14 | 11 | 100.0 | 0.0 | 0.0 | 0.0 |
|  | Salivary duct carcinoma | 15 | 9 | 100.0 | 0.0 | 0.0 | 0.0 |
|  | Acinic cell carcinoma of the salivary gland | 181 | 103 | 99.0 | 0.0 | 1.0 | 0.0 |
|  | Adenocarcinoma NOS of the salivary gland | 109 | 54 | 92.6 | 3.7 | 1.9 | 1.9 |
|  | Adenoid cystic carcinoma of the salivary gland | 180 | 64 | 100.0 | 0.0 | 0.0 | 0.0 |
|  | Basal cell adenocarcinoma of the salivary gland | 25 | 21 | 100.0 | 0.0 | 0.0 | 0.0 |
|  | Basal cell adenoma of the salivary gland | 101 | 58 | 100.0 | 0.0 | 0.0 | 0.0 |
|  | Epithelial-myoepithelial carcinoma of the salivary gland | 53 | 43 | 100.0 | 0.0 | 0.0 | 0.0 |
|  | Mucoepidermoid carcinoma of the salivary gland | 343 | 261 | 99.6 | 0.0 | 0.0 | 0.4 |
|  | Myoepithelial carcinoma of the salivary gland | 21 | 15 | 93.3 | 0.0 | 6.7 | 0.0 |
|  | Myoepithelioma of the salivary gland | 11 | 9 | 100.0 | 0.0 | 0.0 | 0.0 |
|  | Oncocytic carcinoma of the salivary gland | 12 | 6 | 100.0 | 0.0 | 0.0 | 0.0 |
|  | Polymorphous adenocarcinoma, low grade, of the salivary gland | 41 | 21 | 100.0 | 0.0 | 0.0 | 0.0 |
|  | Pleomorphic adenoma of the salivary gland | 53 | 31 | 100.0 | 0.0 | 0.0 | 0.0 |
| **Tumors of the lung, pleura and thymus** | Adenocarcinoma of the lung | 196 | 175 | 5.7 | 3.4 | 3.4 | 87.4 |
|  | Squamous cell carcinoma of the lung | 80 | 66 | 84.8 | 1.5 | 3.0 | 10.6 |
|  | Small cell carcinoma of the lung | 16 | 5 | 20.0 | 0.0 | 0.0 | 80.0 |
|  | Mesothelioma, epithelioid | 40 | 34 | 100.0 | 0.0 | 0.0 | 0.0 |
|  | Mesothelioma, biphasic | 77 | 43 | 100.0 | 0.0 | 0.0 | 0.0 |
|  | Thymoma | 29 | 23 | 60.9 | 26.1 | 4.3 | 8.7 |
|  | Lung, neuroendocrine tumor (NET) | 29 | 24 | 33.3 | 0.0 | 4.2 | 62.5 |
| **Tumors of the female genital tract** | Squamous cell carcinoma of the vagina | 78 | 68 | 97.1 | 1.5 | 1.5 | 0.0 |
|  | Squamous cell carcinoma of the vulva | 157 | 148 | 98.0 | 1.4 | 0.7 | 0.0 |
|  | Squamous cell carcinoma of the cervix | 136 | 131 | 99.2 | 0.0 | 0.8 | 0.0 |
|  | Adenocarcinoma of the cervix | 23 | 23 | 100.0 | 0.0 | 0.0 | 0.0 |
|  | Endometrioid endometrial carcinoma | 338 | 295 | 95.9 | 1.4 | 1.4 | 1.4 |
|  | Endometrial serous carcinoma | 86 | 74 | 91.9 | 5.4 | 0.0 | 2.7 |
|  | Carcinosarcoma of the uterus | 57 | 54 | 90.7 | 5.6 | 1.9 | 1.9 |
|  | Endometrial carcinoma, high grade, G3 | 13 | 8 | 87.5 | 12.5 | 0.0 | 0.0 |
|  | Endometrial clear cell carcinoma | 9 | 6 | 100.0 | 0.0 | 0.0 | 0.0 |
|  | Endometrioid carcinoma of the ovary | 130 | 118 | 97.5 | 0.8 | 0.0 | 1.7 |
|  | Serous carcinoma of the ovary | 580 | 504 | 97.4 | 1.4 | 1.2 | 0.0 |
|  | Mucinous carcinoma of the ovary | 101 | 86 | 95.3 | 2.3 | 0.0 | 2.3 |
|  | Clear cell carcinoma of the ovary | 51 | 46 | 95.7 | 0.0 | 0.0 | 4.3 |
|  | Carcinosarcoma of the ovary | 47 | 47 | 100.0 | 0.0 | 0.0 | 0.0 |
|  | Granulosa cell tumor of the ovary | 44 | 44 | 100.0 | 0.0 | 0.0 | 0.0 |
|  | Leydig cell tumor of the ovary | 4 | 4 | 100.0 | 0.0 | 0.0 | 0.0 |
|  | Sertoli cell tumor of the ovary | 1 | 1 | 100.0 | 0.0 | 0.0 | 0.0 |
|  | Sertoli Leydig cell tumor of the ovary | 3 | 3 | 100.0 | 0.0 | 0.0 | 0.0 |
|  | Steroid cell tumor of the ovary | 3 | 3 | 100.0 | 0.0 | 0.0 | 0.0 |
|  | Brenner tumor | 41 | 41 | 100.0 | 0.0 | 0.0 | 0.0 |
| **Tumors of the breast** | Invasive breast carcinoma of no special type | 499 | 492 | 99.2 | 0.4 | 0.2 | 0.2 |
|  | Lobular carcinoma of the breast | 192 | 189 | 98.9 | 0.5 | 0.0 | 0.5 |
|  | Medullary carcinoma of the breast | 23 | 23 | 100.0 | 0.0 | 0.0 | 0.0 |
|  | Tubular carcinoma of the breast | 20 | 17 | 100.0 | 0.0 | 0.0 | 0.0 |
|  | Mucinous carcinoma of the breast | 29 | 28 | 100.0 | 0.0 | 0.0 | 0.0 |
|  | Phyllodes tumor of the breast | 50 | 46 | 100.0 | 0.0 | 0.0 | 0.0 |
| **Tumors of the digestive system** | Adenomatous polyp, low-grade dysplasia | 50 | 50 | 98.0 | 2.0 | 0.0 | 0.0 |
|  | Adenomatous polyp, high-grade dysplasia | 50 | 49 | 89.8 | 8.2 | 2.0 | 0.0 |
|  | Adenocarcinoma of the colon | 2483 | 2290 | 94.8 | 2.4 | 0.9 | 1.9 |
|  | Gastric adenocarcinoma, diffuse type | 215 | 167 | 100.0 | 0.0 | 0.0 | 0.0 |
|  | Gastric adenocarcinoma, intestinal type | 215 | 188 | 94.1 | 4.3 | 1.1 | 0.5 |
|  | Gastric adenocarcinoma, mixed type | 62 | 60 | 95.0 | 3.3 | 0.0 | 1.7 |
|  | Adenocarcinoma of the esophagus | 83 | 65 | 96.9 | 1.5 | 0.0 | 1.5 |
|  | Squamous cell carcinoma of the esophagus | 76 | 55 | 100.0 | 0.0 | 0.0 | 0.0 |
|  | Squamous cell carcinoma of the anal canal | 91 | 86 | 97.7 | 2.3 | 0.0 | 0.0 |
|  | Cholangiocarcinoma | 121 | 114 | 96.5 | 0.9 | 1.8 | 0.9 |
|  | Gallbladder adenocarcinoma | 51 | 49 | 85.7 | 10.2 | 0.0 | 4.1 |
|  | Gallbladder Klatskin tumor | 42 | 39 | 94.9 | 5.1 | 0.0 | 0.0 |
|  | Hepatocellular carcinoma | 312 | 307 | 98.7 | 1.0 | 0.3 | 0.0 |
|  | Ductal adenocarcinoma of the pancreas | 659 | 624 | 98.2 | 1.0 | 0.5 | 0.3 |
|  | Pancreatic/Ampullary adenocarcinoma | 98 | 96 | 92.7 | 5.2 | 2.1 | 0.0 |
|  | Acinar cell carcinoma of the pancreas | 18 | 18 | 100.0 | 0.0 | 0.0 | 0.0 |
|  | Gastrointestinal stromal tumor (GIST) | 62 | 58 | 100.0 | 0.0 | 0.0 | 0.0 |
|  | Appendix, neuroendocrine tumor (NET) | 25 | 16 | 100.0 | 0.0 | 0.0 | 0.0 |
|  | Colorectal, neuroendocrine tumor (NET) | 12 | 11 | 100.0 | 0.0 | 0.0 | 0.0 |
|  | Ileum, neuroendocrine tumor (NET) | 53 | 51 | 100.0 | 0.0 | 0.0 | 0.0 |
|  | Pancreas, neuroendocrine tumor (NET) | 101 | 93 | 94.6 | 0.0 | 1.1 | 4.3 |
|  | Colorectal, neuroendocrine carcinoma (NEC) | 14 | 12 | 91.7 | 0.0 | 8.3 | 0.0 |
|  | Ileum, neuroendocrine carcinoma (NEC) | 8 | 7 | 85.7 | 0.0 | 14.3 | 0.0 |
|  | Gallbladder, neuroendocrine carcinoma (NEC) | 4 | 4 | 0.0 | 100.0 | 0.0 | 0.0 |
|  | Pancreas, neuroendocrine carcinoma (NEC) | 14 | 14 | 85.7 | 7.1 | 0.0 | 7.1 |
| **Tumors of the urinary system** | Non-invasive papillary urothelial carcinoma, pTa G2 low grade | 177 | 158 | 93.0 | 5.1 | 1.9 | 0.0 |
|  | Non-invasive papillary urothelial carcinoma, pTa G2 high grade | 141 | 117 | 89.7 | 4.3 | 5.1 | 0.9 |
|  | Non-invasive papillary urothelial carcinoma, pTa G3 | 219 | 126 | 97.6 | 0.0 | 2.4 | 0.0 |
|  | Urothelial carcinoma, pT2-4 G3 | 735 | 616 | 97.1 | 1.3 | 0.5 | 1.1 |
|  | Squamous cell carcinoma of the bladder | 22 | 22 | 100.0 | 0.0 | 0.0 | 0.0 |
|  | Small cell neuroendocrine carcinoma of the bladder | 23 | 15 | 26.7 | 0.0 | 6.7 | 66.7 |
|  | Sarcomatoid urothelial carcinoma | 25 | 23 | 95.7 | 4.3 | 0.0 | 0.0 |
|  | Urothelial carcinoma of the kidney pelvis | 62 | 61 | 88.5 | 6.6 | 3.3 | 1.6 |
|  | Clear cell renal cell carcinoma | 1286 | 1224 | 99.9 | 0.0 | 0.1 | 0.0 |
|  | Papillary renal cell carcinoma | 368 | 327 | 99.1 | 0.9 | 0.0 | 0.0 |
|  | Clear cell (tubulo) papillary renal cell carcinoma | 26 | 23 | 100.0 | 0.0 | 0.0 | 0.0 |
|  | Chromophobe renal cell carcinoma | 170 | 151 | 99.3 | 0.7 | 0.0 | 0.0 |
|  | Oncocytoma of the kidney | 257 | 228 | 100.0 | 0.0 | 0.0 | 0.0 |
| **Tumors of the male genital organs** | Adenocarcinoma of the prostate, Gleason 3+3 | 83 | 80 | 100.0 | 0.0 | 0.0 | 0.0 |
|  | Adenocarcinoma of the prostate, Gleason 4+4 | 80 | 71 | 94.4 | 4.2 | 1.4 | 0.0 |
|  | Adenocarcinoma of the prostate, Gleason 5+5 | 85 | 79 | 91.1 | 8.9 | 0.0 | 0.0 |
|  | Adenocarcinoma of the prostate (recurrence) | 258 | 218 | 88.5 | 9.2 | 1.4 | 0.9 |
|  | Small cell neuroendocrine carcinoma of the prostate | 19 | 7 | 28.6 | 0.0 | 0.0 | 71.4 |
|  | Seminoma | 682 | 575 | 100.0 | 0.0 | 0.0 | 0.0 |
|  | Embryonal carcinoma of the testis | 54 | 37 | 100.0 | 0.0 | 0.0 | 0.0 |
|  | Leydig cell tumor of the testis | 31 | 31 | 100.0 | 0.0 | 0.0 | 0.0 |
|  | Sertoli cell tumor of the testis | 2 | 2 | 100.0 | 0.0 | 0.0 | 0.0 |
|  | Sex cord stromal tumor of the testis | 1 | 1 | 100.0 | 0.0 | 0.0 | 0.0 |
|  | Spermatocytic tumor of the testis | 1 | 1 | 100.0 | 0.0 | 0.0 | 0.0 |
|  | Yolk sac tumor | 53 | 43 | 95.3 | 2.3 | 2.3 | 0.0 |
|  | Teratoma | 53 | 39 | 94.9 | 0.0 | 2.6 | 2.6 |
|  | Squamous cell carcinoma of the penis | 92 | 90 | 96.7 | 2.2 | 1.1 | 0.0 |
| **Tumors of endocrine organs** | Adenoma of the thyroid gland | 113 | 107 | 0.0 | 1.9 | 0.9 | 97.2 |
|  | Papillary thyroid carcinoma | 391 | 373 | 0.8 | 0.0 | 0.8 | 98.4 |
|  | Follicular thyroid carcinoma | 154 | 145 | 0.0 | 0.0 | 2.1 | 97.9 |
|  | Medullary thyroid carcinoma | 111 | 96 | 0.0 | 1.0 | 18.8 | 80.2 |
|  | Parathyroid gland adenoma | 43 | 42 | 100.0 | 0.0 | 0.0 | 0.0 |
|  | Anaplastic thyroid carcinoma | 45 | 42 | 81.0 | 2.4 | 2.4 | 14.3 |
|  | Adrenal cortical adenoma | 50 | 38 | 100.0 | 0.0 | 0.0 | 0.0 |
|  | Adrenal cortical carcinoma | 28 | 28 | 100.0 | 0.0 | 0.0 | 0.0 |
|  | Pheochromocytoma | 50 | 50 | 100.0 | 0.0 | 0.0 | 0.0 |
| **Tumors of haemotopoetic and lymphoid tissues** | Hodgkin's lymphoma | 103 | 89 | 98.9 | 1.1 | 0.0 | 0.0 |
|  | Small lymphocytic lymphoma, B-cell type (B-SLL/B-CLL) | 50 | 50 | 100.0 | 0.0 | 0.0 | 0.0 |
|  | Diffuse large B cell lymphoma (DLBCL) | 113 | 113 | 98.2 | 1.8 | 0.0 | 0.0 |
|  | Follicular lymphoma | 88 | 88 | 100.0 | 0.0 | 0.0 | 0.0 |
|  | T-cell non-Hodgkin's lymphoma | 25 | 25 | 96.0 | 0.0 | 0.0 | 4.0 |
|  | Mantle cell lymphoma | 18 | 18 | 100.0 | 0.0 | 0.0 | 0.0 |
|  | Marginal zone lymphoma | 16 | 16 | 100.0 | 0.0 | 0.0 | 0.0 |
|  | Diffuse large B-cell lymphoma (DLBCL) in the testis | 16 | 16 | 100.0 | 0.0 | 0.0 | 0.0 |
|  | Burkitt lymphoma | 5 | 5 | 100.0 | 0.0 | 0.0 | 0.0 |
| **Tumors of soft tissue and bone** | Tendosynovial giant cell tumor | 45 | 16 | 100.0 | 0.0 | 0.0 | 0.0 |
|  | Granular cell tumor | 53 | 29 | 100.0 | 0.0 | 0.0 | 0.0 |
|  | Leiomyoma | 50 | 44 | 100.0 | 0.0 | 0.0 | 0.0 |
|  | Leiomyosarcoma | 94 | 86 | 98.8 | 1.2 | 0.0 | 0.0 |
|  | Liposarcoma | 145 | 105 | 99.0 | 0.0 | 1.0 | 0.0 |
|  | Malignant peripheral nerve sheath tumor (MPNST) | 15 | 14 | 78.6 | 7.1 | 14.3 | 0.0 |
|  | Myofibrosarcoma | 26 | 26 | 100.0 | 0.0 | 0.0 | 0.0 |
|  | Angiosarcoma | 74 | 50 | 100.0 | 0.0 | 0.0 | 0.0 |
|  | Angiomyolipoma | 91 | 89 | 98.9 | 1.1 | 0.0 | 0.0 |
|  | Dermatofibrosarcoma protuberans | 21 | 16 | 100.0 | 0.0 | 0.0 | 0.0 |
|  | Ganglioneuroma | 14 | 12 | 100.0 | 0.0 | 0.0 | 0.0 |
|  | Kaposi sarcoma | 8 | 5 | 100.0 | 0.0 | 0.0 | 0.0 |
|  | Neurofibroma | 117 | 117 | 99.1 | 0.0 | 0.0 | 0.9 |
|  | Sarcoma, not otherwise specified (NOS) | 74 | 71 | 94.4 | 2.8 | 1.4 | 1.4 |
|  | Paraganglioma | 41 | 40 | 100.0 | 0.0 | 0.0 | 0.0 |
|  | Ewing sarcoma | 23 | 18 | 83.3 | 11.1 | 0.0 | 5.6 |
|  | Rhabdomyosarcoma | 7 | 7 | 71.4 | 0.0 | 14.3 | 14.3 |
|  | Schwannoma | 122 | 117 | 58.1 | 10.3 | 12.0 | 19.7 |
|  | Synovial sarcoma | 12 | 9 | 100.0 | 0.0 | 0.0 | 0.0 |
|  | Osteosarcoma | 44 | 27 | 100.0 | 0.0 | 0.0 | 0.0 |
|  | Chondrosarcoma | 40 | 16 | 100.0 | 0.0 | 0.0 | 0.0 |
|  | Rhabdoid tumor | 5 | 5 | 80.0 | 20.0 | 0.0 | 0.0 |
|  | Solitary fibrous tumor | 17 | 17 | 100.0 | 0.0 | 0.0 | 0.0 |
