# Supplementary material for: TTF-1 is a highly sensitive but not fully specific marker for pulmonary and thyroidal cancer: a tissue microarray study evaluating more than 17,000 tumors from 152 different tumor entities
Source: Virchows Arch. 2024 Oct 8;485(5):815–28. doi: 10.1007/s00428-024-03926-1 (PMC11564378; doi:10.1007/s00428-024-03926-1)
Supplement: Supplementary file 4 — Supplementary file4 (DOCX 13 KB) [file 428_2024_3926_MOESM4_ESM.docx]

|  | **TTF-1 immunostaining** | | | |
| --- | --- | --- | --- | --- |
| **Tumor entity** | n | weak (%) | moderate (%) | strong (%) |
| Gallbladder, neuroendocrine carcinoma (NEC) | 4 | 100.0 | 0.0 | 0.0 |
| Lung, neuroendocrine tumor (NET) | 24 | 0.0 | 4.2 | 62.5 |
| Ileum, neuroendocrine carcinoma (NEC) | 7 | 0.0 | 14.3 | 0.0 |
| Pancreas, neuroendocrine carcinoma (NEC) | 14 | 7.1 | 0.0 | 7.1 |
| Colorectal, neuroendocrine carcinoma (NEC) | 12 | 0.0 | 8.3 | 0.0 |
| Pancreas, neuroendocrine tumor (NET) | 93 | 0.0 | 1.1 | 4.3 |
| Appendix, neuroendocrine tumor (NET) | 16 | 0.0 | 0.0 | 0.0 |
| Colorectal, neuroendocrine tumor (NET) | 11 | 0.0 | 0.0 | 0.0 |
| Ileum, neuroendocrine tumor (NET) | 51 | 0.0 | 0.0 | 0.0 |
